# Investigating the impacts of field‐realistic exposure to a neonicotinoid pesticide on bumblebee foraging, homing ability and colony growth

**DOI:** 10.1111/1365-2664.12689

**Published:** 2016-05-30

**Authors:** Dara A. Stanley, Avery L. Russell, Sarah J. Morrison, Catherine Rogers, Nigel E. Raine

**Affiliations:** ^1^ School of Biological Sciences Royal Holloway University of London Egham TW20 0EX UK; ^2^ Botany and Plant Science School of Natural Sciences and Ryan Institute National University of Ireland Galway Ireland; ^3^ Graduate Interdisciplinary Program in Entomology and Insect Science University of Arizona Tucson AZ 85721 USA; ^4^ Lunar and Planetary Laboratory University of Arizona Tucson AZ 85721 USA; ^5^ School of Environmental Sciences University of Guelph Guelph ON N1G 2W1 Canada

**Keywords:** agrochemical, bumble bee *Bombus terrestris*, flower‐visiting insects, insecticide, navigation, neonicotinoids, pesticide exposure, pollination, RFID tagging

## Abstract

1. The ability to forage and return home is essential to the success of bees as both foragers and pollinators. Pesticide exposure may cause behavioural changes that interfere with these processes, with consequences for colony persistence and delivery of pollination services.
2. We investigated the impact of chronic exposure (5–43 days) to field‐realistic levels of a neonicotinoid insecticide (2·4 ppb thiamethoxam) on foraging ability, homing success and colony size using radio frequency identification (RFID) technology in free‐flying bumblebee colonies.
3. Individual foragers from pesticide‐exposed colonies carried out longer foraging bouts than untreated controls (68 vs. 55 min). Pesticide‐exposed bees also brought back pollen less frequently than controls indicating reduced foraging performance.
4. A higher proportion of bees from pesticide‐exposed colonies returned when released 1 km from their nests; this is potentially related to increased orientation experience during longer foraging bouts. We measured no impact of pesticide exposure on homing ability for bees released from 2 km, or when data were analysed overall.
5. Despite a trend for control colonies to produce more new workers earlier, we found no overall impacts of pesticide exposure on whole colony size.
6. *Synthesis and applications*. This study shows that field‐realistic neonicotinoid exposure can have impacts on both foraging ability and homing success of bumblebees, with implications for the success of bumblebee colonies in agricultural landscapes and their ability to deliver crucial pollination services. Pesticide risk assessments should include bee species other than honeybees and assess a range of behaviours to elucidate the impact of sublethal effects. This has relevance for reviews of neonicotinoid risk assessment and usage policy world‐wide.

## Abstract 2

This study shows that field‐realistic neonicotinoid exposure can have impacts on both foraging ability and homing success of bumblebees, with implications for the success of bumblebee colonies in agricultural landscapes and their ability to deliver crucial pollination services. Pesticide risk assessments should include bee species other than honeybees and assess a range of behaviours to elucidate the impact of sublethal effects. This has relevance for reviews of neonicotinoid risk assessment and usage policy world‐wide.

## Introduction

Bumblebees experience their surrounding landscape at large spatial scales (Westphal, Steffan‐Dewenter & Tscharntke 2006a) and can navigate back to their nests from long distances (up to 9·8 km; Goulson & Stout 2001). Foraging ranges for a number of bumblebee species have been estimated using harmonic radar tracking, observational and molecular techniques (Osborne *et al*. 1999; Walther‐Hellwig & Frankl 2000; Knight *et al*. 2005) and range from a few hundred metres to almost 2 km (depending on species and landscape quality; Westphal, Steffan‐Dewenter & Tscharntke 2006b). As bumblebees operate at these large spatial scales, navigation and foraging ability are essential to the foraging success of individual bumblebees. This ability to locate, forage and navigate over large distances from a central nest site in the environment is a cognitively challenging task, and any increased stress on colonies (such as homing failure of individual bees) might lead to colony failure (Bryden *et al*. 2013).

Global bee declines have raised concern over continued provision of pollination services and have been linked with a number of potential factors including the increased agricultural use of pesticides (Brown & Paxton 2009). Pesticides are applied to protect crops from insect pests, but at the same time, non‐target beneficial insects such as bees come into contact with them, although often at sublethal levels (i.e. exposure levels below those reported to have lethal impacts). Neonicotinoids, a group of widely used pesticides, are of particular concern due to their toxicity, systemic properties and application methods (Vanbergen & Initiative 2013; Sanchez‐Bayo & Goka 2014). Treated crops often have neonicotinoid residues in their nectar and pollen, leading to bees coming into oral contact as they forage. Neonicotinoids are neuroactivators that target nicotinic acetylcholine receptors (nAChRs) in the insect brain and can cause neuronal deactivation in the mushroom bodies (Palmer *et al*. 2013), which are brain regions linked with learning and memory (Zars 2000; Menzel 2012). Neonicotinoids have been shown to have a variety of sublethal impacts on honeybees and bumblebees (Godfray *et al*. 2014, 2015), and concern over these sublethal effects has led to a moratorium (Regulation (EU) No 485/2013) on their use on crops attractive to bees in Europe and restrictions in some provinces of Canada.

Although the majority of work has focussed on pesticide effects on honeybees (Godfray *et al*. 2015; Lundin *et al*. 2015), sublethal impacts of field‐realistic neonicotinoid exposure have also been reported for bumblebees, including effects on reproduction (Laycock *et al*. 2012; Whitehorn *et al*. 2012; Elston, Thompson & Walters 2013; Moffat *et al*. 2015; Rundlöf *et al*. 2015), learning ability (Stanley, Smith & Raine 2015), foraging (Feltham, Park & Goulson 2014; Gill & Raine 2014; Stanley & Raine 2016) and delivery of pollination services (Stanley *et al*. 2015). Exposure to field‐realistic levels of imidacloprid caused bumblebee foragers to bring back smaller pollen loads (Gill, Ramos‐Rodriguez & Raine 2012) and pollen less often (Feltham, Park & Goulson 2014). However, not all neonicotinoids have the same toxicity to bees (Mommaerts *et al*. 2010; Moffat *et al*. 2015, 2016), so although bees exposed to thiamethoxam have been shown to behave differently when visiting flowers on their first foraging bout (Stanley *et al*. 2015; Stanley & Raine 2016), nothing is currently known about the impacts of thiamethoxam exposure on foraging ability over the foraging career of individual bees. Pesticide impacts on foraging may be linked with the ability of bees to navigate and return home (Belzunces, Tchamitchian & Brunet 2012; Blacquière *et al*. 2012; Henry *et al*. 2012). Homing encapsulates a range of behaviours that may be affected by pesticides, both cognitive (e.g. memory) and physiological (e.g. metabolism), and as such could be a useful addition to pesticide risk assessments (EFSA 2013; Henry *et al*. 2014). Although impacts of pesticide exposure on homing behaviour and navigation in honeybees have been investigated in both laboratory (Vandame *et al*. 1995; Decourtye *et al*. 2011; Schneider *et al*. 2012; Matsumoto 2013) and field conditions (Henry *et al*. 2012; Fischer *et al*. 2014; Thompson *et al*. 2016a), we know nothing about potential effects of neonicotinoid exposure on homing abilities of bumblebees. Bumblebees are social species with distinctive biology and navigational strategies from honeybees (Osborne 2012), and appear to respond differently in terms of pesticide effects (Cresswell *et al*. 2012; Rundlöf *et al*. 2015). Therefore, it is important to understand how pesticide exposure affects bumblebee homing and foraging ability.

If pesticides cause changes in foraging and homing ability, it follows that colony growth might be affected as reproductive success in bumblebees has been directly linked to food availability (Pelletier & McNeil 2003). Indeed, reduced foraging efficiency of bumblebees exposed to imidacloprid has been shown to result in reduced colony growth (Gill, Ramos‐Rodriguez & Raine 2012; Whitehorn *et al*. 2012), and colonies foraging on clothianidin‐treated fields produced fewer sexuals (Rundlöf *et al*. 2015). Although reproduction of bumblebee micro‐colonies was not affected by thiamethoxam at field‐realistic levels in the laboratory (Laycock *et al*. 2014), and bumblebee colonies next to thiamethoxam‐treated oilseed rape fields developed at a similar rate to control colonies (Thompson *et al*. 2016b), data on how thiamethoxam might affect bumblebee colony growth in the field are lacking.

Our aim was to assess the impacts of chronic exposure to low, field‐realistic levels of a commonly used neonicotinoid pesticide (thiamethoxam) on bumblebee foraging, homing ability and colony growth by asking:

1. Does chronic thiamethoxam exposure affect foraging activity of free‐flying bumblebees?
2. Does chronic thiamethoxam exposure affect bumblebee homing ability?
3. Do any thiamethoxam‐induced changes observed in foraging and/or homing ability result in impacts on colony growth?

We aimed to make our experiment field‐realistic by exposing bees to levels of thiamethoxam that have been measured in pollen and nectar collected by bees in the field (see Appendix S1, Supporting information), using a semi‐field design with colonies located in the laboratory that had unrestricted access to forage on flowers outside (as per methods in Gill, Ramos‐Rodriguez & Raine 2012). We used radio frequency identification tags (RFID) to record the activity of each bee and observed pollen collection of returning foragers (foraging activity), performed releases of individuals at sites 1 or 2 km away from their colonies to examine their ability to return home (homing ability) and observed daily eclosion rates of new bees in each colony (colony growth).

## Materials and methods

Eight commercial *Bombus terrestris* audax colonies were purchased from Biobest in July 2013, each containing a queen and on average 22 workers (range 16–32). All colonies were transferred to bipartite wooden nest boxes, with the brood in one chamber and access to honeybee‐collected pollen and sugar water in the front chamber. On transfer, all individual bees had an RFID tag (mic3‐Tag 16K, Microsensys GmbH, Erfurt, Germany) glued to their thorax. From that point on any newly eclosed workers were recorded and tagged daily.

Colonies were paired with respect to size and amount of brood, and one of each pair was randomly assigned to pesticide or control treatment: resulting in four pesticide‐exposed and four untreated control colonies. Pesticide treatment colonies received a feeder of 40% sucrose solution in the external chamber that contained approximately 2·4 ppb thiamethoxam (dissolved in acetone: range 1·72–2·34 ppb: see Appendix S1), while control colonies received just 40% sucrose solution (containing 2·4 ppb acetone as a solvent control). Colonies received approximately half of their daily intake of artificial nectar, and received no pollen, to stimulate foraging in the external environment (as per Gill, Ramos‐Rodriguez & Raine 2012). Feeders were replenished every Monday, Wednesday and Friday, and workers were tagged daily except for Sunday. All equipment was used with colonies of the same treatment to prevent cross‐contamination.

### Foraging activity

Colonies were placed in the laboratory (51°25′35·68″N 0°33′43·27″W), but could access the surrounding landscape (comprised of the university campus, suburban gardens, parkland and agricultural pasture; Fig. S1). Bees accessed the outside by passing through a pair of RFID readers along a 2‐m tube connected to a hole in the laboratory window with a landing platform outside (as per Gill, Ramos‐Rodriguez & Raine 2012). The order in which RFID readers were activated revealed the direction an individual bee was moving. The window entry/exit holes belonging to each colony were as far apart as possible (0·5–2·5 m apart, half (two per treatment) facing west and half facing north) and uniquely identified with visual landmarks to assist returning individuals to distinguish their own colony and minimize drifting among them.

After 5 days of treatment, we began recording the number of individuals returning with pollen. Each nest was observed for 90 min twice a week – on days when homing trials were not performed – and alternating time of day (morning and afternoon). Four colonies were observed simultaneously per session, and we conducted 11 observation periods per colony.

Custom‐written matlab software was used to process RFID data and extract foraging‐related parameters (see Appendix S2). For each bee we calculated the daily mean number of times they entered the colony (‘visits’), the daily mean number of foraging bouts performed (‘bouts’), the daily mean foraging bout duration and the number of days on which that individual bee foraged. Throughout this manuscript, we define a ‘foraging bout’ as a trip from the colony entrance that lasted more than 5 min and took place during daylight hours, but excluded any trips longer than a day (see Appendix S2 for more details).

### Homing ability

After 2 weeks of treatment exposure, we started homing trials; this ensured that colonies had grown in size and gave foragers sufficient time to fully explore the surrounding environment. Releases were performed from four colonies on 1 day (two per treatment group) and repeated the next day with the remaining four. Releases were limited to warm bright days (average temperature 25 °C, wind speed 7 km h^−1^, rain <0·1 mm). Beginning at 09:30 h, returning bees were caught before re‐entering the nest, their RFID tag read, marked with a colour pen and transferred to a Petri dish with access to an Eppendorf feeder containing a known mass of untreated 40% sucrose solution. This procedure ensured bees could feed to satiation motivating them to return directly to their nest upon release. Feeders were re‐weighed following release to quantify sucrose consumption by each bee. Catching continued until five individuals were collected per colony, or until 13:30 h (whichever was sooner). We measured inter‐tegular width (a body size estimator (Hagen & Dupont 2013), termed ‘body size’ from now on) for each individual using digital callipers. Individuals were released between 13:00 and 15:00 h, resulting in an average of 3 h between when individuals were caught and released. Individuals were released in succession once the previous bee had either flown out of sight or crawled from the Petri dish onto the grass (as bumblebees can learn from each other; Leadbeater & Chittka 2005). The time each bee returned to the colony was recorded by RFID readers. Any returning individuals (colour‐marked) observed within colonies on subsequent days were removed to avoid multiple testing of individuals and to stimulate forager recruitment for future trials. Homing trial release distances alternated weekly between 1 km and 2 km (to provide both an easier and more challenging task, based on pilot tests and Goulson & Stout 2001) for 5 weeks. This resulted in two releases per colony at each distance and 143 individuals released in total. Homing releases at each distance always took place in the same location, and both 1‐km and 2‐km locations were in the same compass direction from the nest location to standardize effects of surrounding landscape (Fig. S1). Prior to release, bees, with an average body size (inter‐tegular) of 5·9 mm, consumed on average 0·125 g of sucrose solution.

We confirmed that individuals released in homing trials consumed treatment solution (sugar water containing pesticide or control) by conducting 100 min of observations per colony at feeders, each week. An RFID pen reader (cling film covered to prevent cross‐contamination among colonies) confirmed that 80% of released bees were observed to feed at the feeder containing treatment solution at least once.

We also performed homing trials following acute pesticide exposure in an additional pilot experiment with low sample size – these data are presented in Appendix S5.

### Colony growth

Colony size was calculated as the number of individuals in the colony at the start of the experiment plus all individuals that eclosed over the course of the experiment, minus any individuals that died or that did not return to the colony (a complete ‘out’ event – see Appendix S2). We measured the inter‐tegular width of a subset of 340 workers at the end of the study to investigate potential treatment effects on the body size of individuals produced.

The experiment ran for 6 weeks, covering the potential exposure period of colonies foraging on oilseed rape that can flower for up to 6 weeks (Stanley & Stout 2014). As the experiment took place in July and August 2013 in a semi‐urban area (Fig. S1), access to pesticide‐treated crops was unlikely as most flower earlier in the season and were not known to occur in the surrounding landscape (at least within 2 km of the study site).

### Data analysis

#### Foraging activity

All statistical analyses were carried out in r version 3.1.0 (R Core Team 2014). We used linear mixed‐effects models, nlme package (Pinheiro *et al*. 2012), to test for differences in the number of visits, average daily number of bouts and mean foraging bout duration per bee between treatments. Data were log transformed (log(*x* + 1)) where necessary to improve the fit of model residuals, and colony membership was included as a random effect. We tested for treatment effects on the number of drifters and foragers by using analysis of variance (anova), and tested for differences in the numbers of bees returning with pollen using linear mixed‐effects models with observation date and colony as random effects.

#### Homing ability

We analysed homing ability in two steps. First, we tested for differences in the number of bees that returned from each distance (1 and 2 km) separately using linear mixed‐effects models, with binomial distribution (specified using GLMER function/lme4 package, Bates *et al*. 2015) and release date nested within colony in the random part. Secondly, using linear mixed‐effects models as described previously, we tested for differences in the time taken for all returning bees to get home for each release distance separately. The amount of nectar bees consumed and their prior foraging experience were included as model covariates, as bumblebee foraging performance improves as they gain experience (Peat & Goulson 2005) with travel distances decreasing 80% between first and last bouts (Lihoreau *et al*. 2012). Prior foraging experience was estimated using the number of days on which each worker had performed foraging bouts prior to the release day (extracted from the RFID data – see Appendix S2). There were no differences in any of these covariates between treatments (body size: linear mixed‐effects model (lme) χ^2^ = 0·21, d.f. = 1, *P *=* *0·65), nectar consumption: lme χ^2^ = 0·15, d.f. = 1, *P *=* *0·70), previous foraging experience (days): lme χ^2^ = 1·48, d.f. = 1, *P *=* *0·22)). However, as body size was positively correlated with nectar consumption (Pearson's product moment correlation; t = 4·19, d.f. = 108, *P *<* *0·001), only nectar consumption was included as a covariate. Models were simplified by removing non‐significant terms, and validated by plotting standardized residuals versus fitted values, normal q‐q plots and histograms of residuals. A number of bees were excluded from homing analyses due to drifting, lack of prior foraging experience and excessive length of time to return (for details see Appendix S3). We also ran a complete model without any data exclusions from both release distances with no covariates (*n* = 143 bees).

#### Colony growth

Treatment differences in colony size, numbers of individuals that left and did not return, number of workers produced and number of dead bees per colony were assessed using general linear models, and data were log transformed (log(*n*) + 1) if necessary to improve model fit. We also tested for differences in body size of a subset of workers that eclosed during the observation period using linear mixed‐effects models with colony as a random effect.

## Results

### Foraging activity

We RFID‐tagged 951 bees of which 558 were recorded leaving the nest (or moving in the tunnels) by RFID readers. We classified 242 (of 558) as foragers (for criteria see Appendix S2), an average of 30 foragers per colony (34 per colony from control and 27 from pesticide colonies). Twenty‐four individuals were found to drift between colonies, with significantly more drifting from natal control than pesticide colonies (anova:* F*_1,6_ = 35·53, *P *<* *0·001, Table 1); these individuals were removed from any further analyses of foraging behaviour as they may have been exposed to both treatments. A total of 86% of foragers foraged for 1–9 days, while the remaining 14% foraged for up to 27 days. Across all foragers, bees performed an average of three foraging bouts per day, each lasting an average of 1 h (range 5–360 min).

**Table 1:** Summary data from foraging activity (a), homing ability (b) and colony growth (c) measurements. Means (± SE) are given, per individual bee or per colony as stipulated. Sample sizes (*n*) of the total number of individuals per measurement are also given, although analyses of colony growth and some of foraging activity were carried out at the colony level (see Materials and methods)

	Control			Pesticide			*P*
	Mean	SE	*n*	Mean	SE	*n*	
(a) Foraging activity							
Number of drifters per colony	5	0·41	20	1·25	0·48	5	***
Number of days foraged per bee	5·22	0·33	135	6·7	0·47	107	ns
Number of foraging bouts/day per bee	2·92	0·18	135	2·87	0·19	107	ns
Number of visits/day per bee	4·37	0·23	135	4·79	0·39	107	ns
Foraging trip duration/day per bee	0·91	0·08	135	1·13	0·09	107	*
Number of foragers per colony	37·5	6·91	150	32·5	7·6	130	ns
Number of foragers returning to colonies	5·74	0·71	241	4·81	0·65	202	ns
Proportion of bees that returned carrying pollen per colony	0·47	0·06	128/241	0·29	0·06	73/202	*
(b) Homing ability							
Proportion of bees that returned 1 km per colony	0·65	0·14	28	0·84	0·12	26	*
Time taken to return 1 km per bee (min)	40·11	9·73	28	60·04	13·71	26	ns
Proportion of bees that returned 2 km per colony	0·34	0·08	24	0·6	0·21	19	ns
Time taken to return 2 km per bee (min)	108·88	39·35	24	57·42	18·98	19	ns
Proportion of bees that returned overall per colony	0·49	0·08	78	0·64	0·14	64	ns
Time taken to return overall per bee (min)	371·13	133·94	78	372·56	118·7	64	ns
(c) Colony growth							
Number of callows emerged per colony	94	7·04	376	99·5	23·1	398	ns
Number of dead bees per colony	15·5	1·66	62	22	6·28	88	ns
Number of bees that did not return per colony	21·5	4·25	86	27·25	10·36	109	ns
Colony size	76	9·68	304	72.25	17·3	289	ns
Body size (mm) per bee	4·07	0·05	130	4·11	0·04	210	ns

*P*‐values show where significant differences between treatments were found, ns = not significant, *P <* *0·05, ***P <* *0·001.

**Figure 1:**
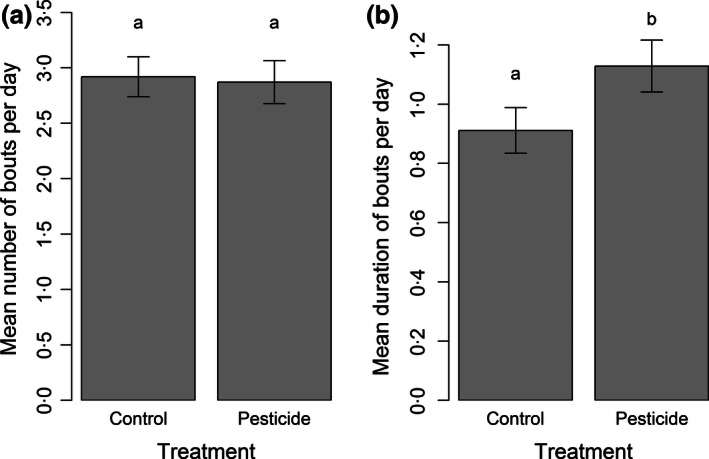
Mean daily number of (a) bouts and (b) bout duration (hours) for bees exposed to control or pesticide (2·4 ppb thiamethoxam) treatments. Columns represent means (± SE) across all individuals recorded as foragers (*n* = 135 individuals in control and 107 pesticide). Letters indicate significant differences (*P *<* *0·05).

We found no treatment differences in the number of days on which bees foraged (glmer: χ^2^ = 1·32, *P *=* *0·25) or the daily number of foraging bouts or visits they performed (bouts: log transformation, lme, χ^2^ = 0·03, *P *=* *0·85; visits: log transformation, lme, χ^2^ = 0·041, *P *=* *0·84; Fig. 1, Table 1). However, we found that pesticide‐exposed bees performed significantly longer foraging bouts (mean ± SE: 68 ± 5 min) compared to controls (mean 55 ± 5 min; log transformation, lme, χ^2^ = 4·01, *P *=* *0·045; Fig. 1). There was also no difference in the number of bees that foraged per colony (anova: χ^2^ = 1·43, *P *=* *0·23).

**Figure 2:**
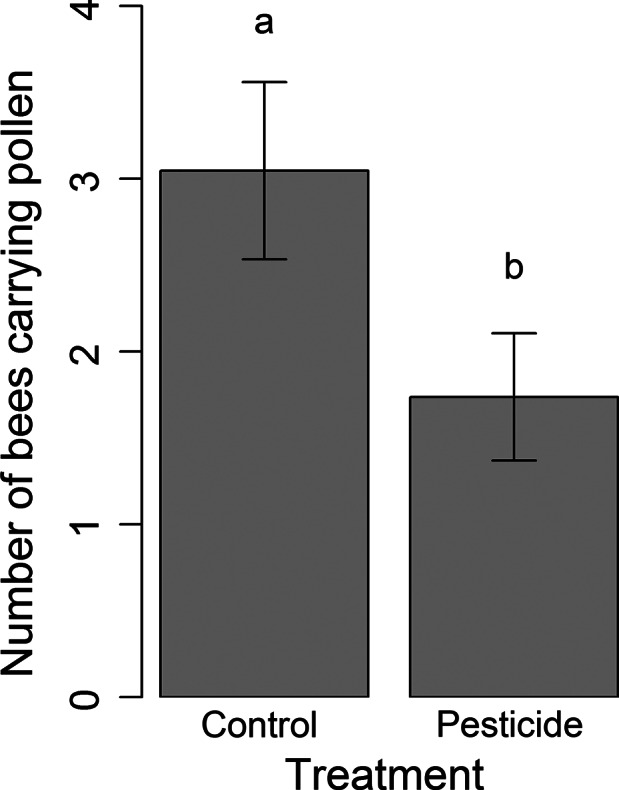
The number of bees returning carrying pollen to colonies exposed to control or pesticide (2·4 ppb thiamethoxam) treatments per observation period (443 returning bees observed in total; of these 128 control bees and 78 pesticide bees carried pollen). Data shown are means (± SE) across four colonies of each treatment on 11 observation days.

While there was no difference between treatments in the numbers of foragers observed returning to colonies (log transformation, lme,: χ^2^ = 0·99, *P *=* *0·32), a greater number of bees returned to control colonies carrying pollen (log transformation, lme: χ^2^ = 4·8, *P *=* *0·03, Fig. 2, Table 1).

### Homing from 1 km

**Figure 3:**
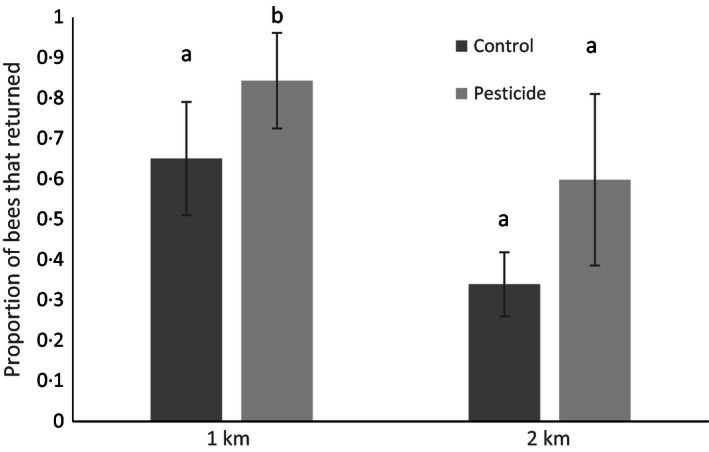
The proportion of bees that returned from each treatment group – control or pesticide (2·4 ppb thiamethoxam) – from release sites at 1 km and 2 km. Data shown are colony means (± SE), although data were analysed at the individual level with colony as a random effect (*n* = 27 control and 26 pesticide‐exposed bees released from 1 km, and 24 control and 19 pesticide‐exposed bees released from 2 km). Analysis showed a significant difference between homing performance of control and pesticide treatments at 1 km, but not at 2 km.

There was a significant impact of treatment on whether bees returned to the nest or not (glmer: χ^2^ = 3·86, d.f. = 1, *P *=* *0·049); 67% (18 of 27 total) of the control bees returned back to their colony, whereas 92% (24 of 26 total) of pesticide‐treated bees returned (Fig. 3, Table 1). The amount of sucrose consumed prior to release was also retained in the best model, with bees that successfully returned to their nest consuming significantly more sucrose (glmer: χ^2^ = 6·3, *P *=* *0·02). For successful bees, the average time taken to return from 1 km was 50 min (range 5–248 min). Although there was a trend for bees exposed to pesticide to take longer to return (mean ± SE; pesticide = 60 ± 14 min, control: 40 ± 10 min), return time was best explained by a model containing only nectar consumption (lme: χ^2^ = 4·33, *P *=* *0·037) – with bees consuming more nectar returning home faster.

### Homing from 2 km

A total of 33% (8 of 24) of bees released returned to control colonies, while 63% (12 of 19) of bees returned to pesticide‐treated colonies (Fig. 3, Table 1). However, treatment was not in the final model explaining homing ability; prior foraging experience levels of bees significantly predicted their ability to return home (glmer: χ^2^ = 4·11, d.f. = 1, *P *=* *0·04). For successful bees, the average time to return home from 2 km was 78 min (range 7–313 min). There was no relationship between time taken to return to the colony and treatment (lme: χ^2^ = 1·72, d.f. = 1, *P *=* *0·19) or any of the other covariates measured (on average, pesticide‐exposed bees took 57 ± 19 min to return home, whereas control bees took 109 ± 39 min; Fig. S2).

We also ran an overall model including all bees that were released over both distances (142 individuals), with no covariates and no individuals excluded based on prior foraging experience, drifting, or time taken to return home (see Appendix S3). Here, we measured no impact of pesticide treatment on whether bees returned home (glmer: χ^2^ = 2·58, *P *=* *0·11), or their time taken to return (lme: χ^2^ = 0·04, *P *=* *0·85, Table 1). However, effect sizes and confidence intervals (Fig. S5) from the releases at 1 and 2 km separately indicate that larger sample sizes may yield differential and opposing impacts of time taken to return at the two release distances.

### Colony growth

**Figure 4:**
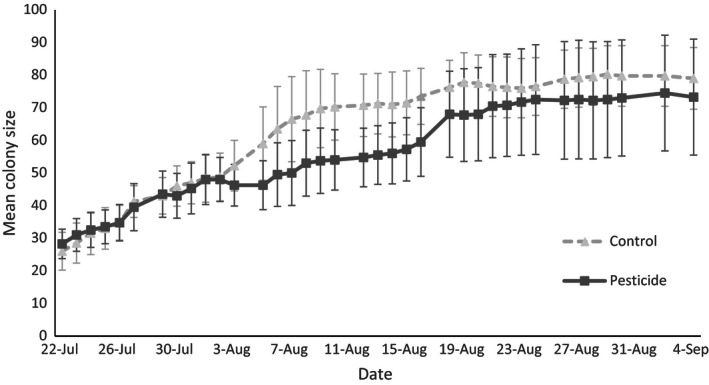
Mean daily size (number of individuals, including number of bees that emerged, number of bees that were found dead in the colony, and number of bees that were recorded leaving the colony without returning) of colonies in each treatment. Data points represent means (± SE) across four colonies in each treatment.

Colony size did not differ between treatments at the start of the experiment (glm: χ^2^ = 0·05, *P *=* *0·82). Over the course of the experiment there was no overall difference in the number of callow workers that emerged (glm: χ^2^ = 0·63, *P *=* *0·43, Table 1), although more callows emerged sooner in control colonies than those exposed to pesticide (Fig. 4). There was no difference in the number of dead bees removed from colonies (glm, logged data: χ^2^ = 0·9, *P *=* *0·77), the number of bees that left their nest but did not return (glm: χ^2^ = 2·72, *P *=* *0·1), overall colony size at the end of the experiment (glm: χ^2^ = 0·38, *P *=* *0·54) or body size of workers produced (lme: χ^2^ = 0·01, *P *=* *0·91; Table 1). However, comparatively large confidence intervals associated with effect sizes for colony growth measurements suggest that larger sample sizes would be needed to increase the robustness of results (see Appendix S4 and Fig. S6).

## Discussion

We found that exposure to low, field‐realistic levels of the neonicotinoid pesticide, thiamethoxam, caused changes in bumblebee foraging patterns, and the proportion of bees that returned home from 1 km. Pesticide‐exposed bees went on longer foraging bouts and collected pollen less often, but found their way back to their colonies from 1 km more frequently during homing trials than bees from control colonies. Although there was a trend for control colonies to produce new workers more quickly than pesticide‐exposed colonies, and more dead bees found inside pesticide colonies, we found no significant impacts of pesticide exposure on overall colony size.

### Foraging activity

The impacts of thiamethoxam on foraging behaviour are consistent with patterns found with imidacloprid; bumblebees exposed to 10 ppb imidacloprid made longer foraging trips and brought back smaller pollen loads (Gill, Ramos‐Rodriguez & Raine 2012), and returned with pollen less frequently after exposure to 0·6 ppb imidacloprid in sugar water and 6 ppb in pollen (Feltham, Park & Goulson 2014). Our work suggests that thiamethoxam‐exposed bees are also less efficient pollen foragers, which has implications for colony development including both worker and sexual production (Pelletier & McNeil 2003). This is also significant for pollination services as bees collecting pollen can be better pollinators for crops and wild plants (Castro *et al*. 2013). In particular, this may be important for plants providing pollen as a sole reward (De Luca & Vallejo‐Marín 2013). Thiamethoxam exposure has been shown to reduce pollination services delivered by bumblebee colonies to apple trees at the start of their foraging career (Stanley *et al*. 2015); our work here shows that pesticide‐exposed bees brought back less pollen over their entire foraging career, suggesting impacts on pollination services may become exacerbated over time.

Perhaps more concerning is that we find impacts of neonicotinoid exposure on bumblebee foraging activity even at very low levels; the thiamethoxam levels we used (2·4 ppb) were below those used previously for imidacloprid. These exposure levels are conservative as we only exposed bees through their artificial nectar source and not pollen, and as bees were free flying, they also had had access to uncontaminated nectar sources in the field.

### Homing ability

The overall proportions of bumblebees found returning to their colonies from 1‐ and 2‐km homing releases was similar to previous work (Goulson & Stout 2001). Although negative impacts of acute thiamethoxam exposure on honeybee homing ability have been documented in a field setting (Henry *et al*. 2012), the levels of pesticide used were higher, and here, we find that a higher proportion of bumblebees exposed chronically to lower levels of thiamethoxam found their way home from 1 km. This result may be partially explained by the different levels of pesticide used. Neonicotinoids are agonists of the acetylcholine receptors and, although they cause neuronal inactivation in the mushroom bodies of honeybee brain (Palmer *et al*. 2013), they can also be partial neural agonists (Déglise, Grünewald & Gauthier 2002) that could result in hormesis (Cutler & Rix 2015). Therefore, another possible explanation for the increased proportion of bees returning to the colony could be that neonicotinoids actually cause ‘excitation’ in other brain regions involved with navigation. Indeed, individual bees exposed to similar levels of thiamethoxam have been shown to visit more flowers than controls in their first foraging bout, although this did not result in increased pollination service delivery as these bees appeared to be behaving differently on crop flowers (Stanley *et al*. 2015).

There could be other behavioural changes that cause this increase in the proportion of bees that return home following exposure to pesticide. For example, as pesticide‐exposed bees went on longer foraging bouts and collected less pollen, they could have spent more time exploring the landscape rather than foraging, making them better able to navigate home. Alternatively, pesticide‐exposed bees may be more prepared to take risks of following either a more direct path home or choosing a flight direction sooner, which under other circumstances (e.g. over longer distances with fewer landmarks) may not be such a successful strategy. Another possible explanation is that control bees were more motivated to forage on the way back to the colony, increasing risks of disorientation and predation. There may also be a selective impact of pesticide; as more dead bees were found in pesticide colonies, it could be that the bees left are the ‘best’ individuals, and therefore are more successful at navigating home. Although we found impacts of pesticide exposure on ability to return, we found no impacts on the time taken to return (similar to work on honeybees: Matsumoto 2013). Time taken was related to nectar consumption, presumably because bees that consumed less nectar (which were smaller) had to stop to forage on the way home.

We released bees at both 1 and 2 km, based on previous studies and pilot work. However, we know bees can forage further afield (Westphal, Steffan‐Dewenter & Tscharntke 2006a) and can return home from much greater distances (Goulson & Stout 2001). Bees exposed to 2·4 ppb thiamethoxam show impaired odour learning and memory performance in the laboratory (Stanley, Smith & Raine 2015); therefore, it could be that the release distances used were relatively unchallenging, and returning from greater distances could be more cognitively difficult. In addition, we released bees on days with optimal weather conditions. As both temperature and solar radiation can influence homing failure in honeybees and, more importantly, as these interact with pesticide effects (Henry *et al*. 2014), the results in our experiment may have been different under less favourable weather conditions. Therefore, it would be interesting to assess bumblebee homing ability in a variety of weather conditions and investigate interactive effects of pesticide exposure.

We have shown that bees can return home from up to 2 km in a relatively short time, but might not complete the task as quickly as they can. The average flight speed of *B. terrestris* has been measured as 7·1 m s^−1^ (Riley *et al*. 1999), with a range of 3–15·7 m s^−1^ (Osborne *et al*. 1999). As our fastest bees took 7 min to return from 2 km, and 5 min from 1 km (equating to speeds of 4·8 and 3·3 m s^−1^ respectively), this suggests that even these bees took some time to orientate rather than travelling immediately back to their colonies. In a similar way to honeybees (Menzel *et al*. 2005), although some individuals flew straight off, the majority of individuals performed orientation flights when released. These exploratory processes may explain the additional time it took bees to return home.

### Colony growth

Previous studies have found impacts of field‐realistic levels of neonicotinoid exposure on bumblebee colony development, including an 85% reduction in queen production (Whitehorn *et al*. 2012), 30% reduction in micro‐colony brood production (Laycock *et al*. 2012), reductions in new worker eclosion rates (Gill, Ramos‐Rodriguez & Raine 2012) and 41 and 71% reductions in male and queen production, respectively (Rundlöf *et al*. 2015). Although we found reductions in eclosion rates of new workers in thiamethoxam‐exposed colonies after a similar period of exposure as Gill, Ramos‐Rodriguez & Raine (2012) – approximately 2 weeks – this was not significant. This time delay is probably related to development; bees that eclosed after this time would have been exposed to pesticide during their development and not just as adults. In comparison with previous work, we may not have found impacts on colony size for three reasons; first, we had a relatively small sample size of four colonies per treatment, which may explain why trends towards higher worker production in control colonies were non‐significant (see Appendix S4 & Fig. S6). Secondly, colonies were of an appreciable size before we began pesticide exposure (average 22 workers). As crops where pesticides are applied often flower early in the season at the very start of the colony cycle, effects on development may be more severe in smaller colonies (e.g. Gill, Ramos‐Rodriguez & Raine 2012). Thirdly, pollen availability has been demonstrated to affect sexual production (Pelletier & McNeil 2003); as pesticide colonies received less pollen, there could have been effects of pesticide on sexual production (Whitehorn *et al*. 2012) which were not apparent over the time‐scale of this study. Alternatively, our work may suggest that thiamethoxam has lower impacts on colony development than imidacloprid. This is supported by Thompson *et al*. (2016b) who found that B. terrestris colonies foraging beside thiamethoxam‐treated oilseed rape developed similarly to controls, and Laycock *et al*. (2014) who, in two separate experiments investigating impacts of imidacloprid and thiamethoxam on fecundity in bumblebee micro‐colonies, also suggest lower impacts of thiamethoxam exposure.

This study shows there are still appreciable sublethal effects on bumblebees, including foraging and homing, at low levels of chronic exposure to a neonicotinoid pesticide. Although we measured no significant impacts on colony size, there may be implications for colony health and reproduction. Either way, our results will be included in the European Food Safety Authority's review of existing evidence to inform the EU moratorium on the use of neonicotinoid pesticides, and has clear policy implications relating to the usage of neonicotinoid pesticides and associated risk of potential harm to pollinators world‐wide. First, we highlight the need to incorporate a range of behaviours, other than reproduction, into risk assessments for neonicotinoids. Secondly, we have shown that bumblebees can be a useful group with which to investigate pesticide effects on pollinator taxa other than honeybees (Osborne 2012), particularly as the severity of effects on honeybees and other bee taxa are frequently not the same (Cresswell *et al*. 2012; Arena & Sgolastra 2014; Rundlöf *et al*. 2015; Piiroinen & Goulson 2016). Thirdly, pesticides are used widely in the environment and our work suggests that a decrease in pesticide use, potentially though integrated pest management, could be beneficial for both pollinating insects and the ecosystem services they deliver. In addition, bees could benefit from pesticide‐free forage as may be provided through untreated crops or wildflower areas.

## Author contributions

DAS & NER designed the experiment, DAS & CR performed the experiment, ALR & SJM developed MATLAB software for processing RFID data, DAS analysed the data, DAS & NER wrote the paper, all authors contributed to revised versions of the paper.

## Data accessibility

Data presented in this paper are deposited in the Dryad Digital Repository http://dx.doi.org/10.5061/dryad.s3j0n (Stanley *et al*. 2016).

## Supporting information


**Fig. S1.** Aerial image of study location.
**Appendix S1.** Rationale for choosing thiamethoxam exposure levels.
**Appendix S2.** RFID data manipulation.
**Appendix S3.** Homing data filtering.
**Appendix S4.** Effect sizes and confidence intervals of colony growth & homing data.
**Appendix S5.** Acute homing experiment data and results.
