# Supplementary material for: Investigating the impacts of field‐realistic exposure to a neonicotinoid pesticide on bumblebee foraging, homing ability and colony growth
Source: J Appl Ecol. 2016 May 30;53(5):1440–9. doi: 10.1111/1365-2664.12689 (PMC5103171; doi:10.1111/1365-2664.12689)
Supplement: Supplementary file 1 — Fig. S1. Aerial image of study location. Appendix S1. Rationale for choosing thiamethoxam exposure levels. Appendix S2. RFID data manipulation. Appendix S3. Homing data filtering. Appendix S4. Effect sizes and confidence intervals of colony growth & homing data. Appendix S5. Acute homing experiment data and results. [file JPE-53-1440-s001.docx]

**Investigating the impacts of field-realistic exposure to a neonicotinoid pesticide on bumblebee foraging, homing ability and colony growth**

Dara A. Stanley^1,2*^, Avery L. Russell^3^, Sarah J. Morrison^4^, Catherine Rogers^1^ & Nigel E. Raine^1,5^

**^1^** School of Biological Sciences, Royal Holloway University of London, Egham, TW20 0EX, UK;

^2^Botany and Plant Science, School of Natural Sciences and Ryan Institute, National University of Ireland, Galway, Ireland;

^3^Graduate Interdisciplinary Program in Entomology and Insect Science, University of Arizona, Tucson, Arizona 85721, U.S.A;

^4^Lunar and Planetary Laboratory, University of Arizona, Tucson, Arizona 85721;

^5^School of Environmental Sciences, University of Guelph, Guelph, Ontario, N1G 2W1, Canada

*email: darastanley@gmail.com

**Supporting Information**

**Contents pg**

**Figure S1** Aerial image of study location 2

**Appendix S1** Rationale for choosing thiamethoxam exposure levels 3

**Appendix S2** RFID data manipulation 4

**Figure S2** Distribution of bouts by time between readers 6

**Figure S3** Distribution of bouts by single reader detections 6

**Appendix S3** Homing data filtering 7

**Figure S4** The average time taken for bees released to return to their nests 7

**Appendix S4** Effect sizes and confidence intervals of colony growth & homing data 8

**Figure S5** Hedges effect sizes and confidence intervals (time taken to return) 8

**Figure S6** Hedges effect sizes and confidence intervals (colony growth) 8

**Appendix S5** Acute homing experiment data and results 9

**References** 10


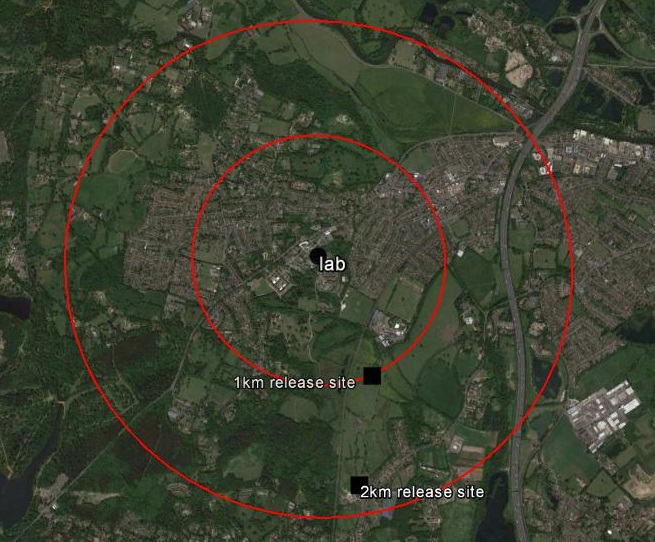
**Figure S1**. Aerial image of the study location, and composition of surrounding landscape (university campus, suburban gardens, parkland and grazing agricultural land) from Google Earth. Red buffer circles indicate radii of 1 km (inner circle) and 2 km (outer circle) from the laboratory in which bumblebee colonies were located (black circle), and both the 1km and 2km release sites for the homing experiment are indicated (black squares). The university campus, where the lab was located, features prominently on the top of a hill, perhaps providing a useful landmark for bees. Map data: Google, DigitalGlobe. Image date 6/6/2013

**Appendix S1. Rational for choosing thiamethoxam exposure levels**

Most studies on the impacts of neonicotinoids on bees have focussed on imidacloprid ([Godfray *et al.* 2015](#_ENREF_5); [Lundin *et al.* 2015](#_ENREF_8)). However, thiamethoxam is another widely used neonicotinoid; for example, it was the most widely applied neonicotinoid as seed dressings on oilseed rape crops in the UK prior to a moratorium on their use ([Garthwaite *et al.* 2012](#_ENREF_4)). Neonicotinoids appear to vary in their toxicity to bees, and in particular thiamethoxam may have less impact on bumblebees than imidacloprid ([Mommaerts *et al.* 2010](#_ENREF_10); [Laycock *et al.* 2014](#_ENREF_7)). Therefore we chose to use thiamethoxam in our work.

The exposure level of thiamethoxam (2.4ppb) was chosen to be field realistic; this concentration has been measured in nectar pots and pollen stores of bumblebee colonies foraging in agricultural areas close to oilseed rape fields ([Thompson *et al.* 2013](#_ENREF_17); [David *et al.* 2016](#_ENREF_3)), in the pollen loads of honeybees returning from neonicotinoid-treated maize fields ([Pilling *et al.* 2013](#_ENREF_12)), in oilseed rape pollen ([David *et al.* 2016](#_ENREF_3)), in the pollen of wild plants growing in agricultural areas ([Botías *et al.* 2015](#_ENREF_1)) and is similar to levels of its metabolite, clothianadin, found in pollen and nectar collected by bees ([Rundlöf *et al.* 2015](#_ENREF_13)). It is also worth noting that these (2.4) ppb calculations are made on a volume per volume basis, while on a mass per mass basis the concentration would be 2ppb.

Three samples of sugar water containing 2.4ppb thiamethoxam that were made up for the experiment were tested for exact thiamethoxam concentration at the FERA lab in York using liquid chromatography - mass spectrometry/mass spectrometry (LC-MS/MS) ([Jones, Harrington & Turnbull 2014](#_ENREF_6)). The range of concentrations measured in the solutions were 1.72-2.34ppb. Therefore, although these measured concentrations were very similar to expected, our results are likely to be conservative as they may indicate average concentrations could have been just below 2.4ppb.

**Appendix S2. RFID data manipulation**

Custom written software (MATLAB R2013b) scripts were developed to automatically process the raw XML data output from the RFID readers (Maja IV reader modules with optimized antenna for mic3-Tag 16K transponders, Microsensys GmbH: Gill *et al.* 2012). Data from 129 individual XML files were automatically combined, whereupon thresholds and scoring criteria (detailed below) were applied to calculate foraging parameters for each bee and exported to Microsoft Excel.

A foraging bout was determined using the following method. An “in” (IN) event was scored as the detection of an RFID tag by the outermost reader (distal to the colony box), of the pair attached to each colony, followed by a detection of that same tag by the innermost reader (proximal to the colony box). Conversely, an “out” (OUT) event was scored as a detection from the innermost reader followed by that from the outermost reader. We set a threshold between inner and outer reader detections (“pairing threshold”) to score whether detections were paired (and therefore constituted a bee leaving or entering the nest as part of a foraging bout). If a bee took longer than 60 seconds to pass through the two readers, the two different reader detections were scored as unpaired. Of the 558 bees detected by both readers (32,120 reader detections), 86.8% of data fell below this 60 second threshold. Below 60 seconds, the mean inter-reader trip duration was 8.37 seconds, with a maximum of 58.9 seconds (Fig. S2). Above the 60 second pairing threshold, the mean inter-reader trip duration was 4017 seconds: this high value was most likely a result of missed detections within a pair of readers.

We scored foraging bouts via two methodologies: firstly, a conservative estimate of a complete foraging bout consists of a bee generating an OUT event followed by an IN event, thus necessitating 4 detections in total (2 detections for each reader pair). We termed this a foraging “bout”. Thus the failure to record any one of these 4 required detections disqualified the putative bout. A complete foraging “bout” was not dependent on colony identity: an OUT event from a reader pair attached to one colony followed by an IN event from a reader pair attached to another colony was scored as a complete bout. Secondly, we also scored foraging bouts using a liberal estimate, based solely on the number of IN events recorded, which we termed a “visit”. The mean duration of foraging bouts recorded using RFID tag monitoring was very similar to values recorded using direct observations of individuals leaving and re-entering nests ([Westphal, Steffan-Dewenter & Tscharntke 2006](#_ENREF_18)).

We scored foraging bouts and visits over the lifetime of each bee. The first and last day of foraging for each bee were excluded when examining patterns of foraging behaviour to ensure our dataset only included days during which bees could forage throughout the day. The duration of a given foraging bout was calculated by determining the time elapsed between paired OUT and IN events (with the average time point of paired reader detections serving as start and end points for a foraging bout). Drifting was automatically scored when an individual bee’s RFID tag was recorded by readers from multiple colonies. We weighted drifting behaviour between colonies for each bee by taking the ratio of its events (INs or Bouts) recorded by each colony’s readers. Numbers of drifters were compared between treatments, using the treatment applied to the natal colony of those drifting individuals.

Each RFID tag can be read multiple times as a bee passes through the reader. We set a threshold for combining sequential same-tag detections (“same bout threshold”). This threshold also affects the number of unpaired detections. For instance, if a bee switches from travelling into the colony to going out of the colony 15 seconds after the last detection from the same reader, a second bout was counted. If a bee took less than 15 seconds to make this manoeuvre, no second bout was counted. Therefore 15 seconds is a conservative threshold to account for bees rapidly unloading the nectar and/or pollen they collected on foraging bouts. Overall 88.3% of the 558 bees detected by readers, (264,774 reader detections), fell below this 15-second threshold (Fig. S3). Below the 15-second threshold, the mean inter-reader trip duration was 0.614 seconds.

Bout duration varied from about 6 seconds to nearly 106 hours. As it is unlikely that very short or long bouts involve foraging, we filtered out both extremes from our dataset using the following thresholds. We excluded all bouts lasting less than 5 minutes (26.0% of bouts or 2125 bouts): these were assumed to be the result of bees exploring the tunnels, making orientation flights, or defecation trips. This threshold was chosen based on pilot observations of how long it took bees to move through the tunnels and fly from the nest entrance, and similar values have been used in previous studies ([Capaldi & Dyer 1999](#_ENREF_2); [Spaethe & Weidenmüller 2002](#_ENREF_14); [Peat & Goulson 2005](#_ENREF_11)). We also excluded bouts that began during the night (defined as 22:00-04:30), as well as bouts that extended into at least a second day, as these bouts were presumed not to be primarily foraging: of the 6055 bouts that lasted longer than 5 minutes, we therefore excluded 9.12% (552 bouts). 528 of these bouts were excluded due to the multi-day threshold and 24 were excluded due to the night-time start threshold. Using these three thresholds we also excluded 15.7% of IN events (“visits”, of 17667 total IN events).

Final outputs were the (i) average number of bouts-, (ii) average number of visits- and the (iii) average duration of bouts per day for each bee. These data were used in assessing the impacts of pesticides on bee foraging behaviour. In addition, we used data calculated on a per day basis to assess the foraging experience of each bee prior to release for the homing experiment, including the total number of days foraging, total number of foraging bouts and visits performed. As these measures were correlated, only the total number of days foraging prior to release was used as a covariate in models exploring pesticide impacts on homing behaviour.

Lastly, we also extracted information on when bees left the colony and did not subsequently return, to feed into data on colony development. We scored leaving without returning via two methodologies: in a liberal estimate, we identified the date of the last reading (paired or unpaired) for each bee. The final detection for 397 of the 607 bees had a paired detection (either IN or OUT). In a conservative estimate, we identified the date of the last OUT event for each bee. The final OUT event for 195 of the 607 bees was not followed by any other events (either paired or unpaired). For statistical analyses of colony development we only reported full OUT events that were not followed by other events.

***Reader failure rate***

Reader failure rate was estimated over 2 hours of observations, involving 45 returning bees. Three of these events failed to be recorded by either reader, while 4 involved only one reader being read. This resulted in 11% of all interactions with individual readers failing (10/90), or 16% of entrance/exits not being fully read (7/45) resulting in a reader failure rate similar to that reported in other studies ([Molet *et al.* 2008](#_ENREF_9); [Tenczar *et al.* 2014](#_ENREF_15)). As treated colonies were distributed evenly between RFID hosts (iID HOST type MAJA 401 dataloggers running iID data capture software MAJA 4.1, Microsensys GmbH), and at distances from the host, it was assumed that reader failures occurred with the same frequency between treatments.


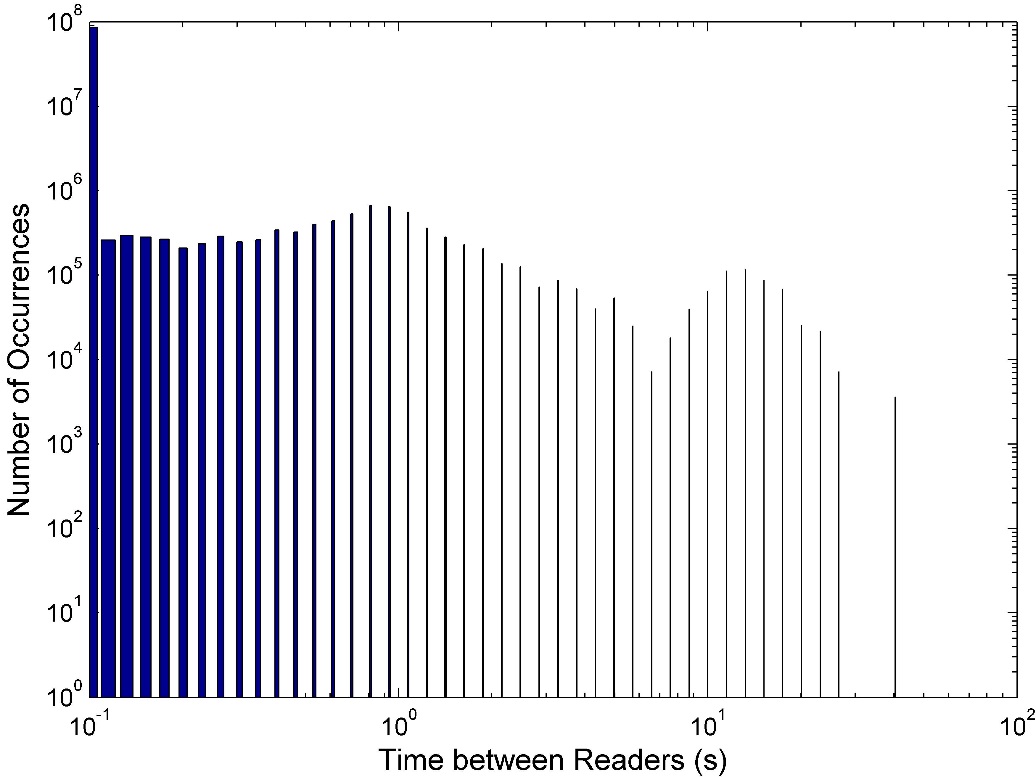


**Figure S2.** Distribution of foraging bouts by the time taken for that individual bee to pass between readers (n = 32,120 reader detections).


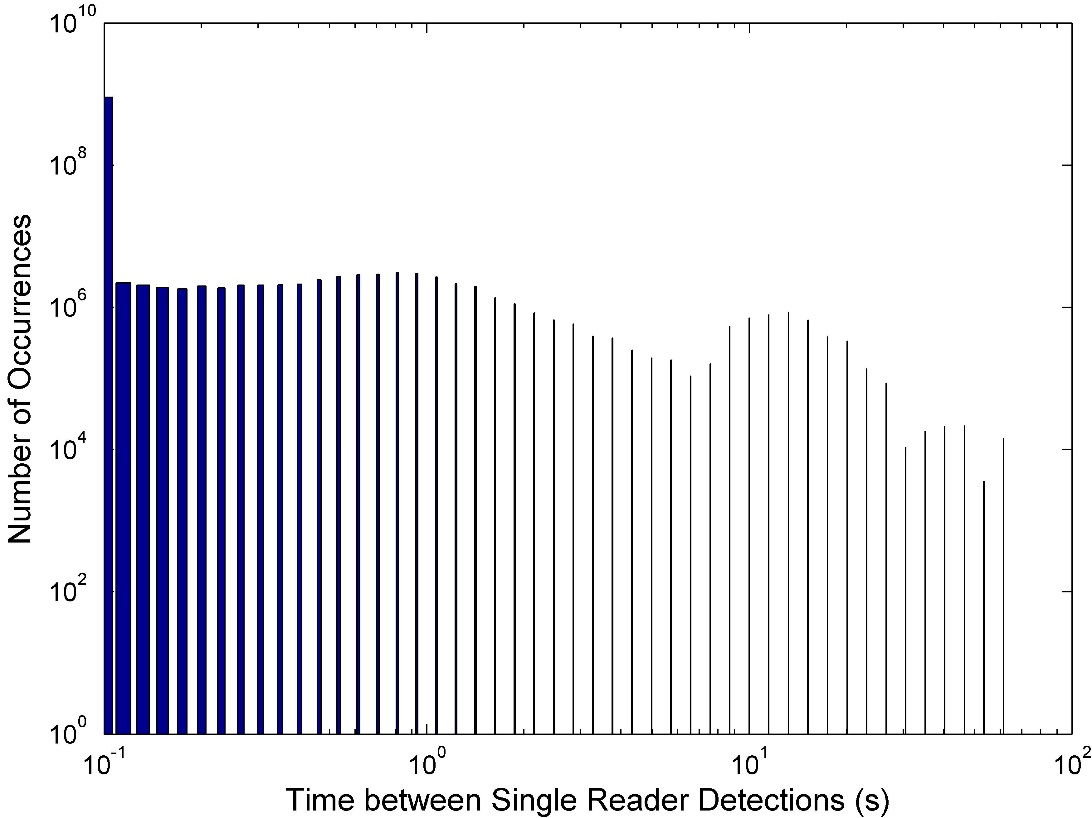


**Figure S3.** Distribution of foraging bouts by the time taken for the same individual bee to be sequentially recorded by the same reader (n = 264,774 reader detections).

**Appendix S3. Homing data filtering**

Bees were excluded from homing analysis if 1) they had no prior foraging experience as any impacts on homing ability caused by treatment could not be discriminated from impacts of experience for these individuals, 2) if they drifted between colonies as they may have been exposed to both treatments and 3) if they took an excessively long time to return home as this homing incidence may have been affected by external factors not related to treatment. To examine the effects of this filtering process, we also ran a full model without any filtered individuals.

*Homing from 1km*

Over the 4 weeks of the experiment, 77 bees were released 1km from their nests. Of these, 12 had no prior foraging experience, 7 drifted between colonies, and 4 bees took between 28 hours and 5 days to return. These individuals were excluded from further analyses, leaving 27 bees from control, and 26 from pesticide treated, colonies.

*Homing from 2 km*

Over the 4 weeks of the experiment, 65 bees were released 2km from their nests. Of these, 7 had no prior foraging experience, 4 drifted between colonies, and 11 bees took 23-96 hours to return; these individuals were excluded, leaving 24 bees from control, and 19 from pesticide, colonies for further analyses.


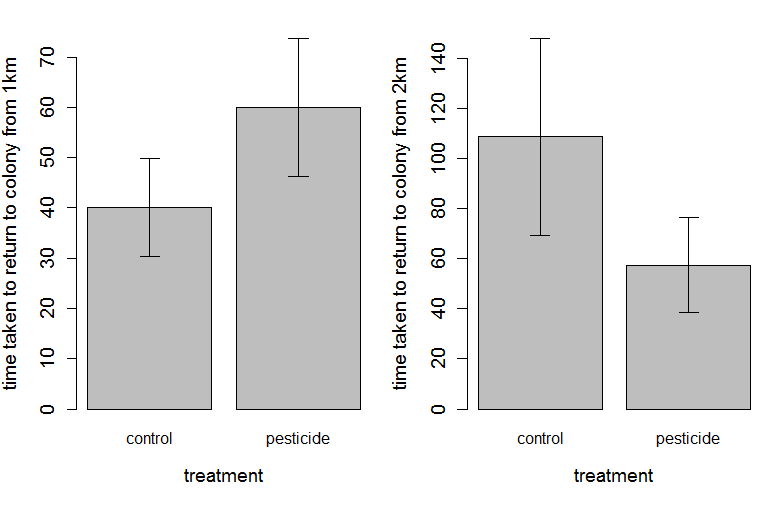


**Figure S4.** The average time taken (in minutes) for bees released 1km and 2km to return to their nests in the chronic homing experiment. Bees were either from colonies exposed to “control” sucrose solution, or colonies chronically exposed to 2.4ppb thiamethoxam (pesticide). Columns show means (± SE) across all individuals released (53 from 1km, and 43 from 2km). There was no significant difference between treatment groups at either distance.

**Appendix S4. Effect sizes and confidence intervals for colony growth and homing measurements**

We examined effect sizes and confidence intervals of colony growth and time taken to return home measurements to infer the power of our data to test pesticide effects ([Thomas 1997](#_ENREF_16)). Although confidence intervals are appreciable for time taken to return home data (Figure S5), confidence intervals barely crossed zero suggesting there may be an opposing treatment effect for 1km and 2km release distances that was not found to be significant here. Although confidence intervals for all data overall are tighter, the difference between release distances appeared to cancel out.

Measurements of colony growth had large confidence intervals that crossed zero (Figure S6). This suggests that we can have relatively low confidence in non-significant results, and that a larger sample size (greater number of colonies per treatment) may be needed to more accurately elucidate the impacts of pesticide treatment.

**Figure S5**. Hedges’ effect sizes and confidence intervals for measurements of time taken to return from either 1km, or 2km and for all data combined (unfiltered data). Overall effect sizes are shown here, although data were analysed with random effects in linear mixed effects models.

**Figure S6.** Hedges’ effect sizes and confidence intervals for colony growth measurements: the numbers of callow workers eclosing (“no. callows”), dead bees and non-returning bees (“no return) per colony and overall colony size.

**Appendix S5. Acute homing experiment**

Methods

In addition to the homing trials performed following chronic thiamethoxam exposure, we also carried out an additional pilot experiment investigating the impacts of acute pesticide exposure on homing ability. These data are not presented in the main manuscript due to small sample sizes. As with the main experiment, we had 8 colonies in the lab with full access to the outside, except this time they received only untreated sucrose solution in their feeders. All methods were the same as in the main (chronic) experiment; however, instead of feeding individuals untreated sucrose solution in the petri dish feeders prior to homing release, we assigned individuals to either “control” or “2.4ppb thiamethoxam” treatments. This ensured bees received a short, acute exposure in the ~3 hours prior to homing release, similar to what they might have received following a single foraging bout in a treated crop field. The experiment ran from mid-September to mid-October 2013.

Results

98 bees were caught and released. 58 of these were from the same colony they were originally tagged in, but 40 were caught entering a different colony indicating drifting was taking place. Overall, the RFID data showed that 62 of these individuals drifted between colonies. Using RFID data, 19 individuals were found to have no previous foraging experience prior to treatment and release (of which none returned); these individuals were excluded from further analyses. A further 18 were did not consume any of their treatment sugar solution or took longer than 17 hours to return; these individuals were also excluded.

There was no difference in foraging experience (F_1,77_ = 0.002, p = 0.96) or nectar consumption (F_1,77_ = 0.006, p = 0.94) of bees assigned to the two treatments. However, bees randomly assigned to pesticide treatments were significantly smaller than control bees (F_1,59_ = 5.8, p = 0.02), presumably an artefact of low sample size.

*Homing from 1km*

Over the 4 weeks of the experiment, 12 bees were released following acute exposure to control (untreated) sucrose solution, while 15 were released following exposure to 2.4ppb thiamethoxam (in sucrose solution). We found no significant difference in the number of individuals that returned between treatments; 67% (8 of 12) individuals returned home following exposure to control, whereas 47% (7 of 15) individuals returned following exposure to pesticide. Although there was no significant difference in the number of bees that returned home, homing ability was correlated to the previous foraging experience of the individual (χ^2^ = 4.44, p = 0.035). There was also no significant treatment difference in the time it took bees to return to the colony (χ^2^ = 0.66, p = 0.42), with control bees taking on average 71 mins to return, and pesticide-treated bees taking 48 mins.

*Homing from 2km*

Of the 18 control bees released 11% (2 of 18) returned home, while only 6% (1 of 17) bees returned following pesticide exposure (this difference was not significant; χ^2^ = 0.26, p = 0.61). This low return rate was surprising. As this experiment was carried out at the end of the flowering season, it could be that although bees were leaving the colony they were not foraging effectively in the environment generating little experience of navigation the area.

**References**

Botías, C., David, A., Horwood, J., Abdul-Sada, A., Nicholls, E., Hill, E.M. *et al.* (2015) Neonicotinoid residues in wildflowers, a potential route of chronic exposure for bees. *Environmental Science & Technology,* **49,** 12731-12740.

Capaldi, E.A. & Dyer, F.C. (1999) The role of orientation flights on homing performance in honeybees. *Journal of Experimental Biology,* **202,** 1655-1666.

David, A., Botías, C., Abdul-Sada, A., Nicholls, E., Rotheray, E.L., Hill, E.M. *et al.* (2016) Widespread contamination of wildflower and bee-collected pollen with complex mixtures of neonicotinoids and fungicides commonly applied to crops. *Environment International,* **88,** 169-178.

Garthwaite, D., Hudson, S., Barker, I., Parish, G., Smith, L., Chippendale, C. *et al.* (2012) Pesticide usage survey report 250: Arable crops in the United Kingdom. York, UK, Food and Environment Research Agency.

Godfray, H.C.J., Blacquière, T., Field, L.M., Hails, R.S., Potts, S.G., Raine, N.E. *et al.* (2015) A restatement of recent advances in the natural science evidence base concerning neonicotinoid insecticides and insect pollinators. *Proceedings of the Royal Society of London B: Biological Sciences,* **282,** 20151821.

Jones, A., Harrington, P. & Turnbull, G. (2014) Neonicotinoid concentrations in arable soils after seed treatment applications in preceding years. *Pest Management Science,* **70,** 1780-1784.

Laycock, I., Cotterell, K.C., O’Shea-Wheller, T.A. & Cresswell, J.E. (2014) Effects of the neonicotinoid pesticide thiamethoxam at field-realistic levels on microcolonies of *Bombus terrestris* worker bumble bees. *Ecotoxicology and Environmental Safety,* **100,** 153-158.

Lundin, O., Rundlöf, M., Smith, H.G., Fries, I. & Bommarco, R. (2015) Neonicotinoid insecticides and their impacts on bees: a systematic review of research approaches and identification of knowledge gaps. *PLoS One,* **10,** e0136928.

Molet, M., Chittka, L., Stelzer, R., Streit, S. & Raine, N. (2008) Colony nutritional status modulates worker responses to foraging recruitment pheromone in the bumblebee *Bombus terrestris*. *Behavioral Ecology and Sociobiology,* **62,** 1919-1926.

Mommaerts, V., Reynders, S., Boulet, J., Besard, L., Sterk, G. & Smagghe, G. (2010) Risk assessment for side-effects of neonicotinoids against bumblebees with and without impairing foraging behavior. *Ecotoxicology,* **19,** 207-215.

Peat, J. & Goulson, D. (2005) Effects of experience and weather on foraging rate and pollen versus nectar collection in the bumblebee, *Bombus terrestris*. *Behavioral Ecology and Sociobiology,* **58,** 152-156.

Pilling, E., Campbell, P., Coulson, M., Ruddle, N. & Tornier, I. (2013) A four-year field program investigating long-term effects of repeated exposure of honey bee colonies to flowering crops treated with thiamethoxam. *PLoS One,* **8,** e77193.

Rundlöf, M., Andersson, G.K.S., Bommarco, R., Fries, I., Hederstrom, V., Herbertsson, L. *et al.* (2015) Seed coating with a neonicotinoid insecticide negatively affects wild bees. *Nature,* **521,** 77-80.

Spaethe, J. & Weidenmüller, A. (2002) Size variation and foraging rate in bumblebees (Bombus terrestris). *Insectes Sociaux,* **49,** 142-146.

Tenczar, P., Lutz, C.C., Rao, V.D., Goldenfeld, N. & Robinson, G.E. (2014) Automated monitoring reveals extreme interindividual variation and plasticity in honeybee foraging activity levels. *Animal Behaviour,* **95,** 41-48.

Thomas, L. (1997) Retrospective power analysis. *Conservation Biology,* **11,** 276-280.

Thompson, H., Harrington, P., Wilkins, S., Piertravalle, S., Sweet, D. & Jones, A. (2013) Effects of neonicotinoid seed treatments on bumble bee colonies under field conditions. York, UK, Food and Environment Research Agency.

Westphal, C., Steffan-Dewenter, I. & Tscharntke, T. (2006) Foraging trip duration of bumblebees in relation to landscape-wide resource availability. *Ecological Entomology,* **31,** 389-394.
